# Supplementary material for: Polydopamine Incorporation Enhances Cell Differentiation and Antibacterial Properties of 3D-Printed Guanosine-Borate Hydrogels for Functional Tissue Regeneration
Source: Int J Mol Sci. 2023 Feb 20;24(4):4224. doi: 10.3390/ijms24044224 (PMC9964593; doi:10.3390/ijms24044224)
Supplement: Supplementary file 1 [file ijms-24-04224-s001.zip › ijms-2169295-supplementary_vMG.pdf]

Supporting Information

Polydopamine Incorporation Enhances Cell Differentiation and Antibacterial Properties of 3D-Printed Guanosine-Borate Hydrogels for Functional Tissue Regeneration

Maria Merino-Gómez, Javier Gil, Roman A. Perez*, Maria Godoy-Gallardo*

Table S1. Scoring table translating the measured values of the measured printability parameters such as collapse area factor (C_f), diffusion rate (D_{fr}), printability (P_r), and angle deviation rate (D_a) into a score ranging from 0 to 40, with 40 being best.

Score	Collapse area factor (C_f)	Diffusion rate (D_{fr})	Printability (P_r)	Angle deviation rate (D_a)
**2**	96 – 100%	96 – 100%	0.00 – 0.05	96 – 100%
**4**	90 – 95%	90 – 95%	0.05 – 0.10	90 – 95%
**6**	85 – 90%	85 – 90%	0.10 – 0.15	85 – 90%
**8**	80 – 85%	80 – 85%	0.15 – 0.20	80 – 85%
**10**	75 – 80%	75 – 80%	0.20 – 0.25	75 – 80%
**12**	70 – 75%	70 – 75%	0.25 – 0.30	70 – 75%
**14**	65 – 70%	65 – 70%	0.30 – 0.35	65 – 70%
**16**	60 – 65%	60 – 65%	0.35 – 0.40	60 – 65%
**18**	55 – 60%	55 – 60%	0.40 – 0.45	55 – 60%
**20**	50 – 55%	50 – 55%	0.45 – 0.50	50 – 55%
**22**	45 – 50%	45 – 50%	0.50 – 0.55	45 – 50%
**24**	40 – 45%	40 – 45%	0.55 – 0.60	40 – 45%
**26**	35 – 40%	35 – 40%	0.60 – 0.65	35 – 40%
**28**	30 – 35%	30 – 35%	0.65 – 0.70	30 – 35%
**30**	25 – 30%	25 – 30%	0.70 – 0.75	25 – 30%
**32**	20 – 25%	20 – 25%	0.75 – 0.80	20 – 25%
**34**	15 – 20%	15 – 20%	0.80 – 0.85	15 – 20%
**36**	10 – 15%	10 – 15%	0.85 – 0.90	10 – 15%
**38**	5 – 10%	5 – 10%	0.90 – 0.95	5 – 10%
**40**	0 – 5%	0 – 5%	0.95 – 1.00	0 – 5%

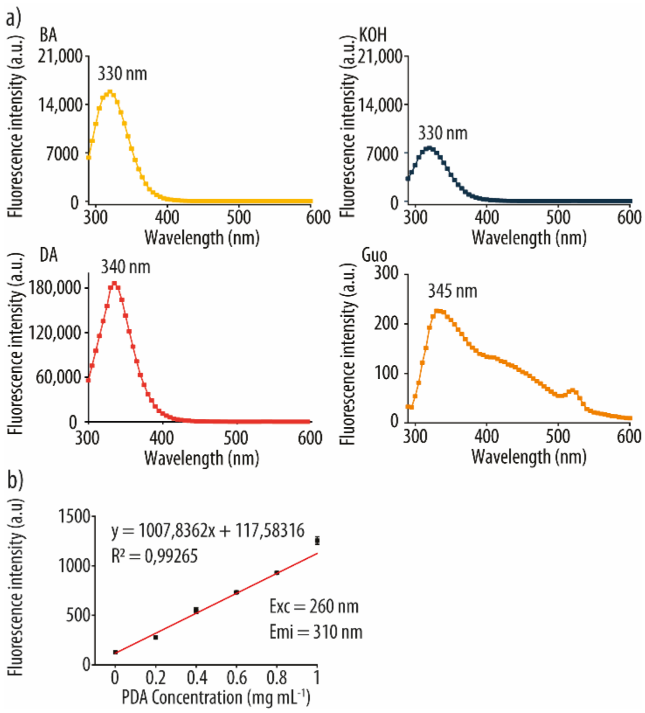

Figure S1. (**a**) Fluorescence intensity scan of boric acid (BA), potassium hydroxide (KOH), dopamine (DA), guanosine (Guo) with excitation and emission wavelength of 260 and 300-600 nm, respectively. (**b**) Calibration curve of Fluorescence intensity if polydopamine (PDA) vs. distinct concentrations of PDA.

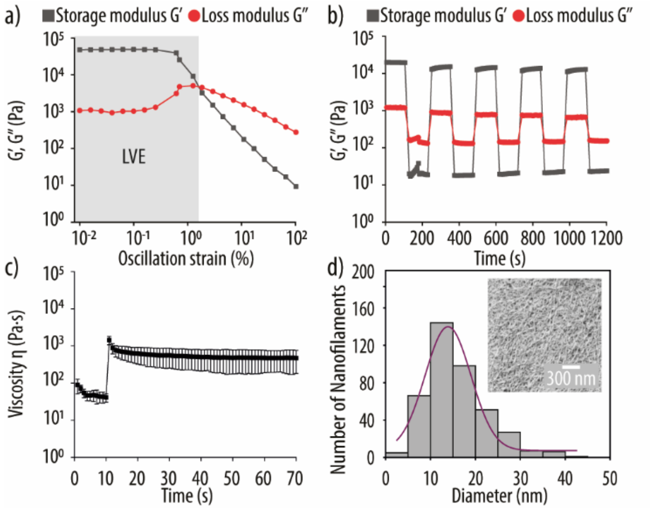

Figure S2. Rheological measurement of guanosine-boric acid (GB) hydrogel at 37 °C. (**a**) Storage modulus (G') and loss modulus (G'') of PGB hydrogels using a strain amplitude sweep (0.1-100%) at a fixed angular frequency (10 rad s^{-1}). Each interval was kept constant for 100 s. (**b**) After applying a shear rate of 3.45 s^{-1} for 10 seconds to mimic the observed forces during 3D printing, we allowed the hydrogel to recover at 0.1 s^{-1} for 60 s. Within ~6 s, optimal hydrogel viscosity was regained. (**c**) Viscosity of PGB hydrogels using a strain range of 0.01-100% and a fixed oscillation frequency of 1 Hz. (**d**) Representative scanning electron microscope (SEM) image of a PGB hydrogel cross-section showing the internal nanofibrillar network and the diameter distribution of the observed nanofibers.
